# The Quantified Brain: A Framework for Mobile Device-Based Assessment of Behavior and Neurological Function

**DOI:** 10.4338/ACI-2015-12-LE-0176

**Published:** 2016-05-04

**Authors:** David E. Stark, Rajiv B. Kumar, Christopher A. Longhurst, Dennis P. Wall

**Affiliations:** 1 Division of Biomedical Informatics, Department of Medicine, Mobilize Center, Department of Bioengineering, Stanford University, Stanford, CA; 2 Department of Pediatrics, Stanford School of Medicine, Stanford, CA, Department of Clinical Informatics, Stanford Children’s Health, Palo Alto, CA; 3 Department of Biomedical Informatics, UC San Diego, La Jolla, CA; 4 Division of Systems Medicine, Department of Pediatrics and Psychiatry (by courtesy), Stanford University, Stanford, CA

## Introduction

Neurological and psychiatric disorders are the greatest cause of disability worldwide [1]. Diagnostic and therapeutic advances have been limited in part due to a paucity of objective and reliable measures of behavior and neurological function – particularly those that are applicable outside the lab or clinic. Mobile devices provide an opportunity to bridge this measurement gap by facilitating unobtrusive longitudinal assessment of complex neurobehavioral states and traits on a population scale. Here we introduce a conceptual framework for innovation that incorporates signals, sensors, and user experience for the measurement of neurological, psychiatric, developmental, and behavioral health (collectively brain health).

## Brain Health’s Measurement Problem

According to recent estimates, brain-mediated neurological and psychiatric disorders account for 28.5% of global years lived with disability (YLDs), more than any other category of disease [1]. Disorders such as depression, anxiety, migraine, epilepsy, dementia, schizophrenia, autism, Parkinson disease, and multiple sclerosis are consistently listed among the major contributors to worldwide disability. It is well recognized that behavioral and mental health are also important determinants of overall health, with behavioral and other preventable factors accounting for 48% of premature deaths in the U.S. -- outstripping genetic, environmental, or healthcare-related factors [2].

Despite substantial progress in neuroscience, few gains have translated into morbidity and mortality improvements. This is due in large part to our inability to precisely measure or define the very diseases under study. Paradoxically, as we develop increasingly sophisticated tools and techniques to observe and quantify invisible processes including gene expression, protein function, and neural activity, we remain unable to reliably measure function and behavior at the organism level. Instead, we continue to rely on subjective assessments and signs-and-symptoms based classifications of disease that are lacking in biological validity. This inability to quantify behavioral patterns (or phenotypes) leaves our research studies underpowered to recognize small effect sizes and our clinicians illequipped to detect and diagnose disease. In the assessment of brain health, there is a critical unmet need for quantitative measurement tools.

## Landscape for change

Patient-generated health data is now a core component of modern healthcare. In his 2015 State of The Union address, President Obama announced the Precision Medicine Initiative calling for “creative new approaches for detecting, measuring, and analyzing a wide range of biomedical information,” and “new models for doing science that emphasize engaged participants.” Over half of this $215 million investment is dedicated to the creation of a voluntary national research cohort of over a million Americans who will contribute their personal health data, including mobile device and sensor data, to the cause [3].

Shortly after this announcement, Apple unveiled ResearchKit -- a framework that enables smartphone users to voluntarily participate in research studies by sharing their mobile device data. Within two weeks of the launch, 30,000 people had already signed up for Stanford’s cardiovascular disease study, one of five pilot programs [4]. At the same time, the Centers for Medicare and Medicaid Services (CMS) and the Office of the National Coordinator for Health IT (ONC) announced proposed criteria for mandating collection of patient-generated health data by the year 2017 [5].

Where will mobile health (or mHealth) prove most impactful? While the Precision Medicine Initiative is initially focused on oncology, and mHealth applications currently leading the charge are focused on cardiovascular disease and diabetes, mHealth may ultimately achieve its greatest gains by taking aim at understanding, diagnosing, and treating disorders of the human brain. Consumer technologies are well positioned to quantitatively assess real world function and behavior, as we discuss below.

## A Brain Health Innovation Framework

In the sections that follow, we propose a conceptual framework to guide mobile device innovation for assessment of brain health. Our framework (see figure) comprises three key dimensions: signal (what is measured), sensor (how it is measured), and user experience (the interaction between the user and the sensor). Technologies that will facilitate the most meaningful gains will arguably engage the upper bounds along each of these dimensions; that is, innovations that enable direct measurement of objective signals using ubiquitous sensors in a passive and continuous fashion.

Why the need for a framework? Given the national effort now underway to implement and scale the vision provided by the Precision Medicine Initiative, we are likely to see an increasingly diverse set of stakeholders involved, spanning the technical, health, and policy domains. Against this backdrop, a conceptual model provides a common language and construct for communicating and testing new ideas [6]. Additionally, our framework aligns with the Food and Drug Administration’s (FDA) stated goal of involving patients as partners throughout the lifecycle of medical research and product development [7]. Collection of patient-generated health data via existing mobile devices enables patient involvement at all stages, from early phase studies, through validation, clinical translation, and continual refinement.

## Signal

Signal refers broadly to anything that conveys information about an underlying behavior or physiological process. To derive value from mHealth tools, we must redirect our focus from subjective categorization of disease states toward quantitative measurement of more objective signals. Whether for research or clinical use, neurological function is currently most often characterized by patient or clinician reporting of signs and symptoms via surveys, rating scales, or clinical history and examination. Gold standard measures like the Beck Depression Inventory, Clinical Dementia Rating Scale (CDR), Unified Parkinson Disease Rating Scale (UPDRS), and Expanded Disability Status Scale (EDSS) for multiple sclerosis achieve clinical utility predominantly due to their test-retest and interrater reliability but they lack biological validity [8, 9].

Measurement of objective signals can complement and augment traditional measures to enable disease prediction, stratification, and treatment selection. For example, in autism, eye tracking has been used to identify abnormalities in infants between the ages of 2–6 months, substantially earlier than the diagnosis is typically made currently [10]. Similarly, measurement of heart rate variability has been used to identify combat soldiers at increased risk for Post-Traumatic Stress Disorder (PTSD) and target them for additional treatment [11]. Vocal analysis has been used to subtype Parkinson disease patients by disease severity [12, 13]. In many neurological disorders including stroke, multiple sclerosis, and Parkinson disease, device-based motion sensors are already being used to assess and monitor function on an ongoing basis. It is neither necessary nor desirable to develop objective clinical measures that attempt to replace their subjective counterparts on a one-to-one basis. Rather, a set of objective measures, in concert with more traditional assessment tools, may provide multiple dimensions on which to map the phenotypic space of behavior and brain function in fine detail.

## Sensor

A sensor provides the means by which a signal is detected and transduced into something usable. The development, validation, and deployment of novel clinical measures stands to benefit greatly from the use of widely-available consumer devices instead of more specialized hardware. The neuroscience research community has had considerable success in characterizing behavior and neurological function on multiple levels from molecules to circuits, in large part due to the refinement of techniques and tools including electroencephalography (EEG) and functional magnetic resonance imaging (fMRI) that enable noninvasive recording of neural activity in the lab. However, to fully realize the benefits of this work, this molecule-to-circuit level understanding of neurological function must be complemented by equally detailed understanding of human behavior and function at the organism level, ideally in the naturalistic ‘real world’ setting. To develop tools for achieving such understanding, innovators should look to sensors that are already in real world use.

90% of the world’s population is projected to own a mobile phone by 2020, with an average of 7 connected devices per person [14]. 93% of smartphone owners keep their device within 3 feet, twenty-four hours per day. The average smartphone user checks their device over 150 times per day [15]. This omnipresence offers a compelling opportunity to deploy measurement tools via the devices that people use on a regular basis.

Beyond mobile phones, other consumer-oriented technologies (particularly those developed for communications, gaming, and user interface control) may be repurposed as biomedical sensors as their use becomes more widespread. Kinect, an optical motion capture system released by Microsoft for its gaming console in 2010, has since been studied extensively in the biomedical setting. To date, over 400 Kinect-related papers have been indexed in MEDLINE [16, 17], and Microsoft Research and Novartis have recently announced the joint development of a Kinect-based tool for assessment in multiple sclerosis [18]. While such work is nascent and requires clinical validation, this approach to device-based behavioral and neurological assessment demonstrates the advantages of using existing consumer devices.

Patient recruitment and retention is often the rate-limiting step in clinical studies, and in clinical practice, patient engagement (particularly in behavioral health interventions) is a significant challenge. For both obstacles, consumer devices are promising tools because they are already integrated within daily routines and facilitate passive data collection with no significant increase in patient activation energy.

## User Experience

User experience encompasses all aspects of the interaction between the user and the sensor(s). In developing novel clinical measures, innovators should strive for passive measurement. This is important not only for driving user adoption and engagement, but also for enabling unobtrusive and continuous measurement of trends or fluctuations in behavior and neurological function over time. Brain health assessments often suffer from an observer effect in which an individual’s performance is modified in response to being observed. Passive measurement enables longitudinal assessment of behavioral and neurological states, and minimizes such measurement bias.

A nascent but rapidly growing body of literature now exists to support the feasibility and utility of passive monitoring of brain health. ‘Smart home’ and wearable sensors have been used to passively identify the onset of mild cognitive impairment and latent Alzheimer disease [19] and to differentiate healthy seniors from those with Parkinson disease [20]. Automated analysis of transcribed free speech has successfully predicted later onset of psychosis in at-risk young adults [21]. Mobile phone usage patterns (frequency and duration of app use, texting, emailing, voice communications, and motion) have been studied to detect mood instability associated with depression, bipolar disorder, and schizophrenia, and to automatically alert users and their care providers to concerning changes [22–24].

Additional opportunities to passively measure behavior and function will emerge as sensors are increasingly embedded in everyday objects and new modes of human-device interaction are deployed. For example, engineers have developed EEG electrodes that can be worn continuously in the ear similar to a hearing aid or mobile headset [25]. ‘Connected toothbrushes’ now on the market boast 3D motion sensors to monitor brushing habits. Automobile insurance providers already offer “pay as you drive” plans that adjust in response to sensor-derived driving patterns.

## Challenges

Challenges facing development and adoption of novel mHealth measures can be divided into three broad categories:

## Technical

Collection and integration of data across different devices and platforms entails a need for standards to ensure interoperability. Analysis requires robust statistical methods to deal with noisy, sparse, irregularly sampled data and feature engineering oriented toward time series data. Both sets of challenges are being addressed by groups spanning academia, industry, non-profits, and international standards organizations [26–31].

## Methodological

The question of how to validate novel brain health measures is of central importance. One approach is simply to evaluate against existing gold standards. Yet, when existing standards themselves lack validity or do not exist, validation can pose a quandary [8]. Alternatively, we can assess putative measures on their ability to identify biologically homogeneous subpopulations or clusters. If these clusters are stable across different cohorts, are linked to primary clinical outcomes (e.g. feeling, function, survival) and map to other biomarkers, we will have affirmation to move forward toward clinical translation.

## Ethical, Legal, and Sociocultural

The following challenges deserve more attention than space allows and are thoroughly covered elsewhere, therefore the interested reader is encouraged to follow the referenced links.

The regulatory landscape surrounding mHealth innovation is rapidly evolving, leading to inevitable confusion that may pose a barrier to innovation. The FDA issues periodic guidance on mobile medical applications (for the latest information visit: http://goo.gl/fqVqpv).

Data security and privacy constitute the greatest challenges facing mHealth innovation, particularly in a domain as sensitive as psychiatric and neurological health. Of the 600 most commonly used mHealth apps, only 30% were shown to have a privacy policy [32]. The recent hacking and randsomware attacks on several hospital systems demonstrate the reality that sensitive electronic health data are vulnerable to a wide array of threats. The FDA oversees digital health cybersecurity (see: http://goo.gl/RpjXev).

Substantial uncertainty and confusion has surrounded the application of federal privacy regulations to patient-generated health data and mobile medical apps under the Health Insurance Portability and Accountability Act (HIPAA). This recently led the Department of Health and Human Services (HSS) to issue specific guidance on the matter (see: http://goo.gl/mhSz7u).

The engagement of patients as partners in development and evaluation of device-based measures presents opportunities as well as challenges due to the potential to blur the lines between consumer, research participant, and patient, and will require discussion to adequately protect participants without hampering innovation. In recognition of these challenges, the FDA has partnered with academia and industry to form the Medical Device Innovation Consortium focused on medical device regulatory science [7]. Similarly, HSS and fifteen other federal departments and agencies are working together to revise the Common Rule governing protection of human research subjects [33]. The goal of such discussions is to strengthen privacy and security safeguards while reducing unnecessary regulatory burden and enabling secondary research on nonsensitive data collected for other purposes.

## Conclusion

We have described a simple framework that can be used to guide effective and impactful innovation within the mobile health space. Pressed with a need for quantitative measurement of behavior and neurological function, innovators should canvas the opportunity space comprised of signal, sensor, and user experience in search of tools possessing the optimal characteristics (ideally those supporting objective, passive, ubiquitous measurement of brain health). With participation of patients throughout the process from development to validation and deployment, we believe that novel brain health measures can be rapidly tested and translated into clinical use.

It is anticipated that other frameworks will emerge as innovators gain experience developing within this unique space. We hope our framework serves as a useful sounding board for further discussion, and expect that it will be adapted and modified as needs evolve.

## Figures and Tables

**Fig. 1:**
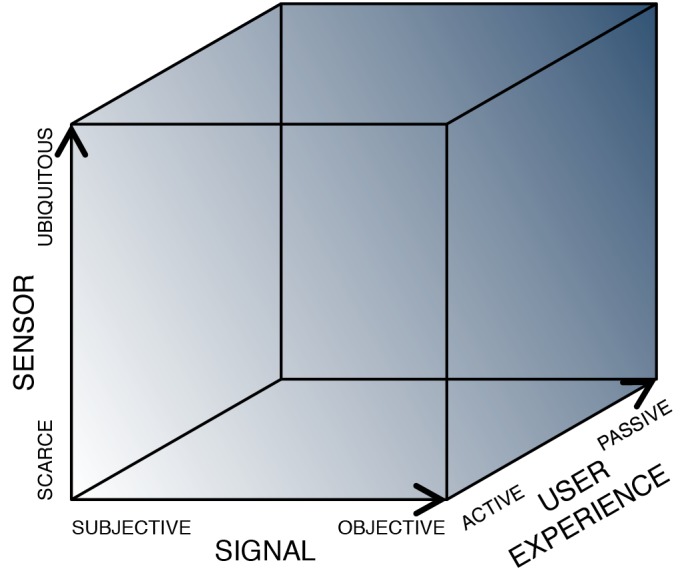
Opportunity space for mobile-device based assessment of behavior and neurological function.
